# Microbiota isolate collections: A key to global vector-borne disease control

**DOI:** 10.1371/journal.pbio.3003078

**Published:** 2025-03-18

**Authors:** Miguel Medina Muñoz, Holly L. Nichols, Kerri L. Coon

**Affiliations:** Department of Bacteriology, University of Wisconsin-Madison, Madison, Wisconsin, United States of America

## Abstract

Manipulation of insect vector microbiota is a promising strategy for controlling vector-borne diseases. This Perspective outlines how microbial isolate collections aid in identifying microbial targets, and why their implementation must rely on coordinated international efforts that are ethical and ensure equitable benefit sharing.

## Teaser

Insect vector microbiomes are essential for insect function, making them promising targets for controlling vector-borne diseases. Microbial isolate collections aid in identifying microbial targets, but expanding these collections must rely on coordinated efforts that are ethical and ensure equitable benefit sharing.

Vector-borne diseases are a growing threat. The precipitous increase in dengue cases in 2024, alongside the emergence of Oropouche virus, continued invasion of malaria-transmitting *Anopheles stephensi* in Africa, and predicted habitat expansion of insect vectors due to global warming, highlight the urgency of controlling vector-borne disease [1]. The use of insecticides, e.g., in insecticide-treated bed nets to repel and kill mosquitoes, is a common method for preventing spread of vector-borne diseases such as malaria. However, insecticides may have off-target effects, persist in the environment, and contribute to pollution; resistance has also repeatedly evolved against many insecticide classes and in various vector species [2]. Alternative strategies therefore need to be considered.

Like humans, insects have their own microbiomes, and manipulation of microbiota (i.e., the constituent members of the microbiome) to control vector-borne diseases is an emerging technology that leverages microorganisms already present in nature, with minimal (if any) engineered modifications to improve accuracy and control persistence. There are examples of this approach, including the World Mosquito Program, which successfully deploys *Wolbachia*-infected male mosquitoes to decrease local dengue cases [3,4]. Additional techniques in development include experimental designs targeting the kissing bug vector of Chagas disease with symbiotic *Rhodococcus rhodnii* engineered to produce molecules toxic to the trypanosome parasite [5], using *Bacillus* to prevent *Leishmania* colonization in the sand fly vector of Leishmaniasis [6], and employing nanobody-producing symbiotic *Sodalis* in the tsetse fly vector of sleeping sickness [7].

To select the most suitable microorganism(s) for vector control, understanding the microbial ecology within the insect is paramount; in addition to understanding the identity of microbes across a vector insect’s geographic distribution and life cycle, knowledge of how these microbes are acquired and transmitted within a population is necessary for implementing successful and long-lasting microbe-based vector control strategies. However, for widely distributed insect pests, such as the yellow fever mosquito, *Aedes aegypti*, discovering omnipresent patterns of microbe–host associations and interactions requires systematically sampling their microbiota across the entire habitat range. Sampling natural habitats is ideal as they represent the actual host–microbe background faced by pathogens that colonize the vector. However, this approach has disadvantages, including the extensive ranges to cover, the adverse and inaccessible landscape features in vector habitats, and legal and ethical constraints that emerge when vector populations overlap human habitation.

An alternative strategy is to capitalize on existing libraries owned by specialized laboratories that have already carried out microbiota collection efforts for their own purposes. The advantages include preprocessed samples with associated metadata, ease of transport and exchange, and fostering collaborative relationships within the field. However, heterogeneity of sample collection methods due to inherent experimental design biases can complicate statistical inferences and isolates that originate from laboratory host colonies may not represent a natural physiological or microbiological background due to inbreeding or controlled rearing conditions.

To overcome these limitations, we recently created the Mosquito Associated Isolate Collection (MosAIC), which aims to catalog, preserve, and study mosquito microbiota diversity [8]. Microbiota collections allow the discovery of adaptation signatures and, in the case of host-associated microorganisms, enable a reductionist approach to study the effect of individual taxa and defined communities on host biology under controlled conditions. The importance of microbiota isolate collections has been recognized due to the involvement of bacterial flora in basic physiological functions of numerous eukaryotic models, including humans [9], pigs [10], cows [11], mice [12], and black soldier flies [13]. Indeed, collections for human-associated microbiota already exist, including initiatives like the Microbiota Vault and Global Microbiome Conservancy project, which aim to safeguard microbial diversity by creating global repositories for microbial samples. These collections also support research in agriculture, helping develop sustainable pest management strategies and improve soil health. In medicine, microbiota collections are essential for studying the human microbiome, leading to advancements in understanding diseases, developing probiotics, and creating personalized medicine approaches. Now, the MosAIC is the first collection launched specifically to help identify microbial candidates for insect vector control, providing a comprehensive repository of microbial isolates and whole genome assemblies from various mosquito species collected from diverse geographical locations [8].

**Fig 1:**
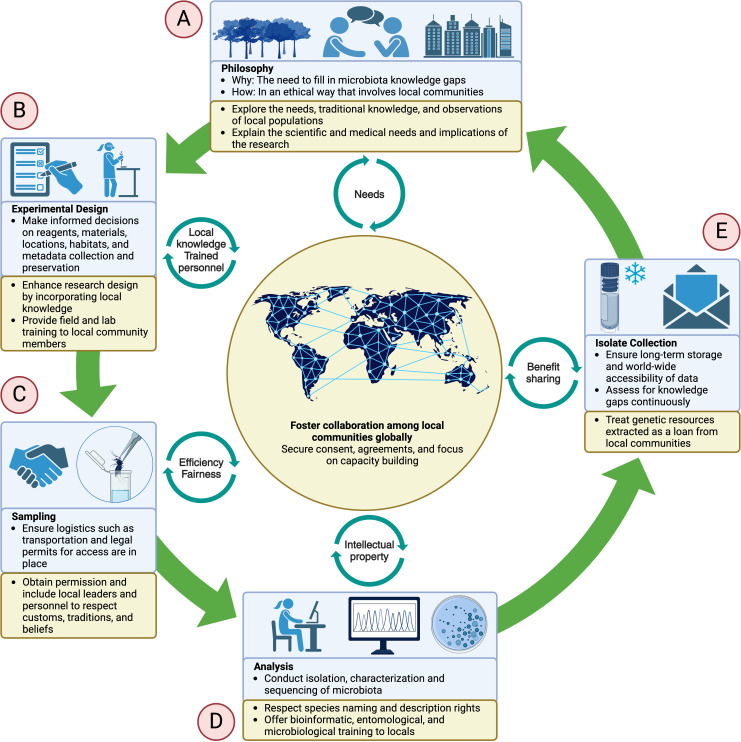
Critical factors involved in the establishment and expansion of microbiota isolate collections. (A) Foundational principles guiding the creation and maintenance of microbiota collections, including the importance of ethical considerations, equitable resource sharing, and adherence to legal frameworks such as the Nagoya Protocol. Principles should underscore the collaborative spirit necessary for successful global partnerships and the commitment to transparency and community engagement. (B) The need for meticulous planning and standardization in the methodologies used to collect and study microbial isolates. This includes the importance of defining clear objectives, selecting appropriate controls, and ensuring reproducibility through standardized protocols. Detailed metadata recording to provide context and enhance the utility of the collected data is also essential. (C) Strategies for systematic sampling across different habitats and life stages of host organisms, along with the challenges associated with accessing remote or legally restricted areas and the importance of obtaining community consent and cooperation. (D) Methods used to analyze collected microbial isolates, including techniques for microbial identification, genomic sequencing, and data interpretation. Robust bioinformatics tools and statistical methods are needed to draw meaningful conclusions from the data and for the potential discovery of novel microbial functions and interactions that can inform vector control strategies. (E) Practical aspects of maintaining and expanding microbiota isolate collections, including the importance of preserving microbial diversity through proper storage techniques, and the role of isolate collections in facilitating research and innovation. Successful examples, such as the MosAIC, and the potential for these collections to drive advancements in microbiome research and global health, are noted. Figure created with BioRender.com.

However, international cooperation on microbiota collections does not come without its challenges. Historically, the extraction of biological resources from low- and middle-income countries, particularly in the global South, has been plagued by unfair practices that often benefit one party and prove detrimental to the other. In recent years, high-income countries have developed an increased awareness of the unethical and unsustainable nature of past approaches. Consequently, global regulations have emerged that set the foundations for equitable participation in the sharing of biological resources and the outcomes derived from their research (Fig 1A).

The Nagoya Protocol (https://www.cbd.int/abs/doc/protocol/nagoya-protocol-en.pdf) provides a working framework for building trustworthy research collaborations among participating countries. It is based on the pillars of informed consent prior to the extraction of genetic resources, mutual agreement on the rules governing the extraction, and the sharing of the benefits derived from every step of the research project. Bringing on board personnel with experience in regulations such as the Nagoya Protocol must be a starting point for the successful establishment of globally sourced microbiota isolate collections, as has been the case with the Global Microbiome Conservancy Project and the MosAIC.

Universal guidelines need to be in place regarding experimental and ethical considerations to maximize the value, accessibility, and acceptance of microbiota collections and translational applications derived from them. Experimentally, conditions for sampling need to be standardized (Fig 1B), such as minimum number of samples, materials, methods, and reagents used for sample collection and preservation, as well as metadata recording such as habitat description, life stage, season, location, and physiochemical parameters. Procedures for microbial and host species identification need to be agreed upon. Additionally, housing a copy of these collections in endemic areas can enhance collaboration with local researchers and promote equity in scientific research, ensuring that benefits derived from these collections are shared with the communities most affected by vector-borne diseases.

Ethically, having permission and engagement from local communities is essential. Approaching communities will vary depending on local customs, but general principles should be followed at different stages. First, a sincere effort must be made to obtain approval before sampling starts (Fig 1C) to avoid violating rules, traditions, or beliefs. Second, incorporating local talent (Fig 1D) is ideal for ensuring the project’s efficiency and endurance. Local collaborators are the best observers of vector population features due to their immersion in vector habitats and proximity to the burden of vector-borne diseases; they develop a unique perspective that will improve research when incorporated in design and execution stages. Recognizing existing capacities and providing resources to grow local research infrastructure will empower communities served by the research. In this way, local collaborators and communities become increasingly invested in stewardship towards local genetic resources and research outcomes. Third, transparency towards the community regarding funding and supporting agencies is crucial. As funds are likely to come from government projects ultimately paid for by taxpayers, transparency is crucial to create a sense of ownership in the communities. Lastly, community approval is necessary before deploying any translational application derived from vector microbiota research. This can be achieved by building trust with local leaders and through planned information campaigns. Ideally, this approach should be complemented with ways to give back to participating communities and institutions, such as providing educational materials, talks, infographics, or interactive content, or openly sharing data with interested parties, such as regional medical and research centers. Acknowledging the benefit-sharing guidelines outlined in the Nagoya Protocol as a starting point, it is crucial to continue the conversation on ensuring that vector control derived from biological resources truly benefits the resource owners. Efforts should consider whether information sharing is sufficient, if financial returns should go back to the community, and the ethics of profiting from biological resources in the context of vector control and public health.

In summary, vector borne diseases are a global threat that require a coordinated response. Microbiota collections facilitate accessibility and exchange of experimental resources across research institutions globally. This exchange serves as a stepping stone for multidisciplinary collaborations that will be instrumental in generating translational applications (Fig 1E) aiming to improve human health across countries. Thus, the establishment of accessible microbiota collections has great potential to generate outcomes that align with the principles of global health [14].
